# Echocardiography protocol: A tool for infrequently used parameters in mice

**DOI:** 10.3389/fcvm.2022.1038385

**Published:** 2022-12-21

**Authors:** Emily Ann Todd, Monique Williams, Ali Kamiar, Monica Anne Rasmussen, Lina A. Shehadeh

**Affiliations:** ^1^Department of Medical Education, University of Miami Leonard M. Miller School of Medicine, Miami, FL, United States; ^2^Department of Medicine, University of Miami Leonard M. Miller School of Medicine, Miami, FL, United States; ^3^Interdisciplinary Stem Cell Institute, University of Miami Leonard M. Miller School of Medicine, Miami, FL, United States

**Keywords:** echocardiography, mitral regurgitation, mitral stenosis, pulmonary regurgitation, aortic regurgitation, cardiovascular disease, left ventricular outflow tract, right ventricular outflow tract

## Abstract

Echocardiography is frequently used to evaluate cardiac function in rodent models of cardiovascular disease. Whereas methods to acquire the commonly used echocardiography parameters are well-described in published protocols or manuals, many important parameters are ill-defined and often open to subjective interpretation. Such lack of uniformity has engendered conflicting interpretations of the same parameters in published literature. In particular, parameters such as mitral regurgitation, mitral stenosis, pulmonary regurgitation, and aortic regurgitation that are required to define more esoteric etiologies in rarer mouse models often remain equivocal. The aim of this methods paper is to provide a practical guide to the acquisition and interpretation of infrequently used echocardiography parameters and set a framework for comprehensive analyses of right ventricle (RV), pulmonary artery (PA) pulmonary valve (PV), left atrium (LA), mitral valve (MV), and aortic valve (AoV) structure and function.

## 1 Introduction

Echocardiography is a non-invasive imaging modality and a pillar of preclinical cardiovascular research. Rodent models for human disease are routinely analyzed *via* a common set of parameters that are limited to <30% of those possible from modern echocardiographic instrumentation. The remaining ∼70% of parameters that are often essential for comprehensive and accurate characterization of murine cardiovascular pathology have not to our knowledge been formally standardized and the relevant procedures are absent from current protocols and manuals of rodent echocardiographic imaging technology. While there are examples of uncommon parameters being acquired to expand on a pathology, details of the acquisition procedures used for such parameter is often lacking. Here we provide an easily accessible protocol of infrequently used views and parameters that will allow for more complete analyses of the right ventricle (RV), pulmonary artery (PA), pulmonary valve (PV), left atrium (LA), mitral valve (MV), and aortic valve (AoV).

## 2 Materials and equipment

The images acquired in this paper were captured using Vevo 2100 and 3100 imaging systems and analysis with Vevo Lab, however, the methods described may be applicable to other multi-modal imaging systems.

### 2.1 Methods

Cardiac function and morphology were assessed using either Vevo 2100 or 3100 imaging systems with a high frequency transducer probe MS400 (VisualSonics, Toronto, ON, Canada). The anterior chest and abdomen of mice were depilated using Nair depilatory cream (Church and Dwight Co., Ewing, NJ, USA) 1 day before experiments (1). On the day of image acquisition, mice were anesthetized with 2.5–3.0% isoflurane at 0.8 L/min flow rate and maintained with 1–1.5% isoflurane (1). After anesthesia, mice were restrained in a supine position on a pad with heater, temperature sensor and ECG electrodes (1). Temperature and heart rate were maintained at 37°C and maximum of 500 beats per minute, respectively (1, 2).

#### 2.1.1 Parasternal long axis view

**FIGURE 1:**
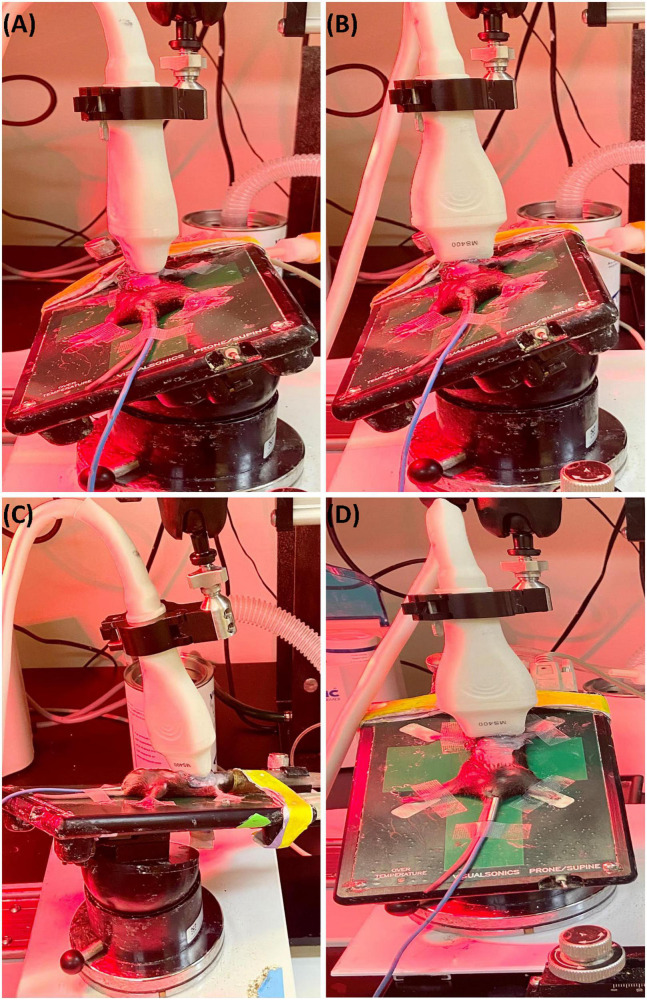
**(A)** Representative image showing probe positioning to obtain parasternal long axis (PLAX) views. **(B)** Parasternal short axis (PSAX) views are derived from the PLAX view by rotating the transducer probe in the rail system to approximately 90° clockwise. **(C)** To obtain apical views, the transducer probe is placed diagonally toward the head. **(D)** Views of the aorta can be acquired by shifting the transducer probe from the PSAX view cranially to the level of the aortic valve.

To obtain parasternal long axis (PLAX) views, the mouse is placed in a supine position with the right side of the platform inclined at 45° counterclockwise (Figure 1A), and the transducer probe placed in the rail system oriented diagonally 30–40° clockwise from the right upper extremity to the left abdomen.

#### 2.1.2 Parasternal short axis view

The parasternal short axis view (PSAX) is derived from the PLAX view by rotating the transducer probe in the rail system to approximately 90° clockwise (Figure 1B).

#### 2.1.3 Apical view

To obtain apical views, the platform is placed slightly lateral with the head of the mouse pointing to the left, and the right upper side of the platform positioned 30° counterclockwise. The transducer probe is placed diagonally oriented toward the head (Figure 1C).

#### 2.1.4 Aorta

With the anterior portion of the platform tilted 45° superiorly and slightly to the left, the transducer probe is shifted from the PSAX view cranially to the level of the aortic valve (Figure 1D).

### 2.2 Right-sided parameters

#### 2.2.1 Right ventricle

Right-sided heart assessment is important for evaluating right heart failure (RHF) and pulmonary hypertension in rodent models, but the possible methodologies are underused in mice despite the availability of clinical guidelines for such imaging (2). Higher frequency, higher resolution, and smaller transducers have permitted advancement of RV imaging in mice despite difficulties related to the morphology and retrosternal position of the RV (2, 3). Given the difficulty in obtaining an apical four chamber view in mice and due to the frequent visual obstruction of the RV by the ribs and sternum in the parasternal short axis (PSAX), the parasternal long axis (PLAX) is the preferred echocardiographic window.

##### 2.2.1.1 Right ventricular internal diameter

**FIGURE 2:**
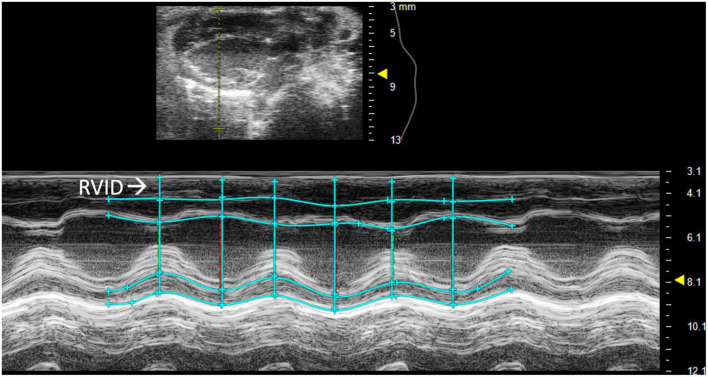
Right ventricular internal diameter (RVID), a measurement of the right ventricle during systole and diastole, is obtained by analyzing parasternal long axis (PLAX) view images in M-mode.

Right ventricular internal diameter (RVID) provides metric dimensions of the right ventricle during systole and diastole. To obtain this parameter, a PSAX or PLAX M-mode image should be captured with the M-mode line positioned over the RV. Obtaining the correct view may require repositioning the platform 45° to the right. Furthermore, skin reverberation artifacts can easily be confused with the RV, therefore flow on Doppler or movement in a B-mode image should be used to confirm that the RV is correctly identified. In PSAX or PLAX M-mode, RVID;s and RVID;d should be selected [instead of the intraventricular septum (IVS) IVS;s and IVS;d] to measure the diameter of the right ventricle (Figure 2). This parameter will also measure IVS, left ventricular internal diameter (LVID), and left ventricular pulmonary wall (LVPW). Increased RVID would be indicative of right ventricular dilation or volume overload and thus useful to evaluate RHF.

##### 2.2.1.2 Right ventricular outflow tract length

**FIGURE 3:**
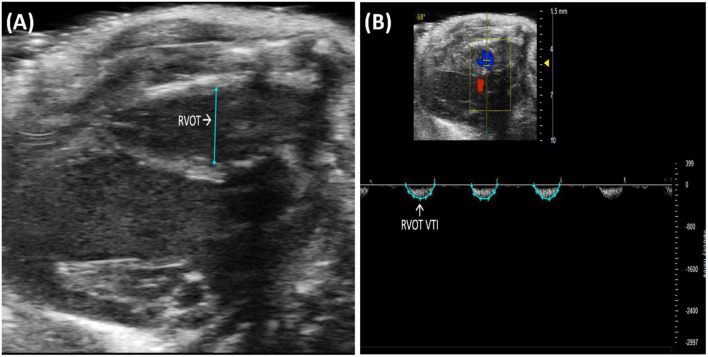
**(A)** Right ventricular outflow tract (RVOT) measures the size of the right ventricular outflow tract at the end of diastole and is acquired and analyzed in B-mode of the parasternal long axis (PLAX) view. **(B)** Right ventricular outflow tract velocity time integral (RVOT VTI) measures blood flow through the right ventricular outflow tract (RVOT) and is acquired by pulsed wave (PW) Doppler imaging of the RVOT in B-mode of the parasternal long axis (PLAX) view.

Right ventricular outflow tract (RVOT) length is the measurement of the right ventricular outflow tract at end diastole. It can be measured by selecting “RVOT” in a PLAX B-mode image containing the RV (Figure 3A). The RVOT is located just proximal to the PV. RVOT is another indicator of RV size (2); a dilated RVOT suggests right ventricular volume overload.

##### 2.2.1.3 Right ventricular outflow tract velocity time integral

Right ventricular outflow tract velocity time integral (RVOT VTI) is a measurement of blood flow through the RVOT. It can be measured as the area under the curve in a pulsed wave (PW) Doppler image of PLAX with the yellow marker on the flow of the RVOT (Figure 3B). Mean velocity, mean gradient, peak velocity, and peak gradient are automatically calculated. RVOT VTI is a surrogate for RV stroke volume (SV), hence, the ratio of RVOT VTI to pulmonary artery systolic pressure represents pulmonary arterial compliance, an important measure for pulmonary arterial hypertension (PAH) (4). Right ventricular systolic pressure is equal to pulmonary artery systolic pressure (5) and can be measured by right heart catheterization in mice. RVOT and RVOT VTI can be used to calculate right ventricular SV and cardiac output (CO).


$$R\,V\,O\,T\,S\,V=0.785\times R\,V\,O\,T^{2}\times R\,V\,O\,T\,V\,T\,I$$

$$R\,V\,O\,T\,C\,O=\frac{R\,V\,O\,T\,S\,V\times H\,R\,f\,r\,o\,m\,R\,V\,O\,T}{1000}$$

#### 2.2.2 Pulmonary artery and valve

**FIGURE 4:**
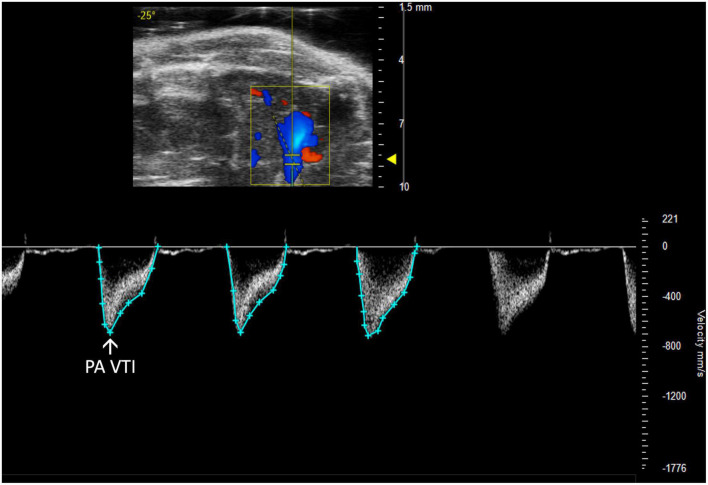
Pulmonary artery velocity time integral (PA VTI) directly measures blood flow through the pulmonary artery and is acquired as the area under the curve in pulsed wave (PW) Doppler mode of the parasternal long axis (PLAX) view.

**FIGURE 5:**
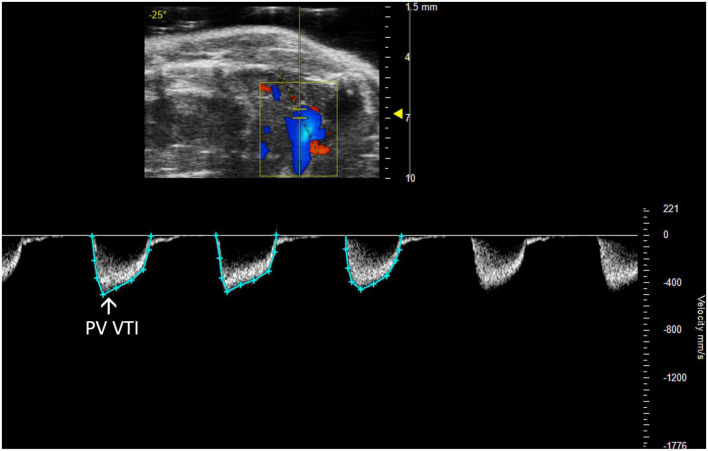
The pulmonary valve velocity time integral (PV VTI), a measure of blood flow through the pulmonary valve, is calculated by measuring the area under the curve in pulsed wave (PW) Doppler mode of the parasternal long axis view (PLAX).

Pulmonary and right ventricular parameters are more informative when applied concurrently. RV dysfunction in the absence of elevated PA pressures indicates isolated RHF, while RV dysfunction concurrent with elevated PA pressures could indicate pulmonary hypertension leading to RHF or LHF leading to RHF. PA and PV measures may be incorrectly assumed to be interchangeable; however, the PA is anatomically distal to the PV in mice, and images should be captured accordingly. Measurements are made of PW Doppler mode images with the marker overlying either the PA or PV (Figures 4, 5).

##### 2.2.2.1 Pulmonary artery velocity time integral

Pulmonary artery velocity time integral (PA VTI) is a direct measure of blood flow through the pulmonary artery. It is calculated as the area under the curve in a PW Doppler image of the PA (Figure 4). Mean velocity, mean gradient, peak velocity, and peak gradient are automatically generated. This measure can be used to evaluate PAH because increased PA flow accompanies PAH (6).

##### 2.2.2.2 Pulmonary regurgitation peak velocity

**FIGURE 6:**
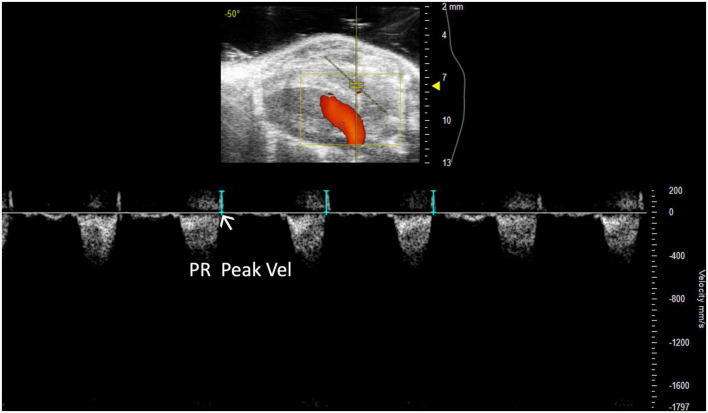
Representative image displaying pulmonary regurgitation peak velocity (PR Peak Vel), a parameter used in mouse models of pulmonic regurgitation which is acquired in pulsed wave Doppler mode of the parasternal long axis (PLAX) view.

Pulmonary regurgitation peak velocity (PR Peak Vel) relates to degrees of pulmonic regurgitation in mouse models. PR Peak Vel is the maximum velocity of the regurgitant stream. Like most other pulmonary measures it is performed with a PW Doppler mode image of pulmonary artery flow (Figure 6). This measure allows for classification of the severity of the regurgitation.

##### 2.2.2.3 Pulmonic valve diameter

**FIGURE 7:**
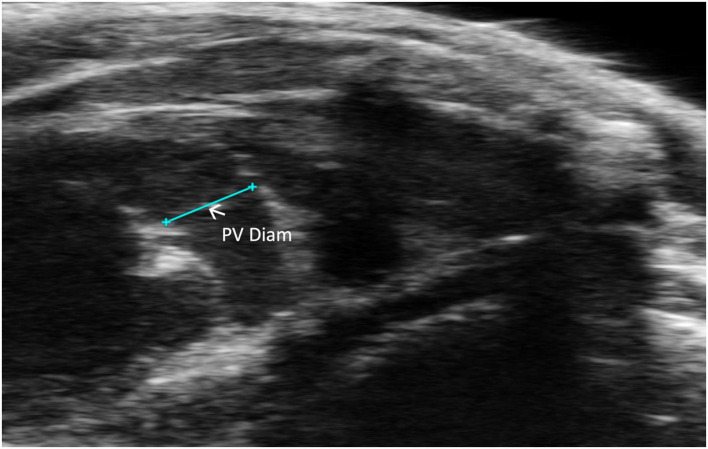
Pulmonic valve (PV) diameter is acquired and analyzed in B-mode of the parasternal long axis (PLAX) view.

Pulmonic valve diameter (PV diameter) measures, in millimeters, the length of the pulmonic valve. This measurement can be performed using a PLAX or PSAX view of the pulmonic valve; however, visualization is typically easier in PLAX (Figure 7). PV diameter can be used to assess whether pulmonic valve stenosis or dilation is present. The PV diameter can also be used with PV VTI to calculate PV SV and CO.


$$P\,V\,S\,V=0.785\times P\,V\,d\,i\,a\,m^{2}\times P\,V\,V\,T\,I$$

$$P\,V\,C\,O=\frac{P\,V\,S\,V\times H\,R\,f\,r\,o\,m\,P\,V\,d\,i\,a\,m}{1000}$$

### 2.3 Left-sided parameters

#### 2.3.1 Left atrium

Left atrial size is rarely evaluated in small animal echocardiography despite the reciprocity between LA reservoir function and LV systolic function and thus cardiac output (7). In other words, LA pathology, leading to decreased preload affects CO and LV systolic function. Furthermore, the LA can be used as a marker of chronic LV dysfunction (8) as is influenced by elevated left ventricular end diastolic pressure (LVEDP) propagating backwards (9).

##### 2.3.1.1 Left atrial depth

**FIGURE 8:**
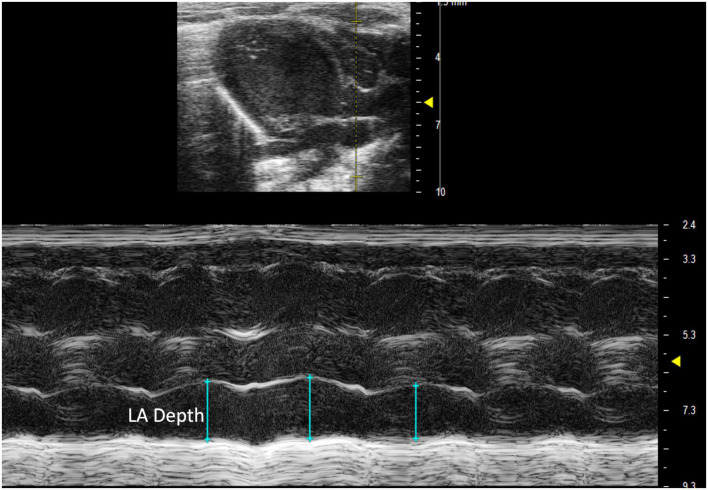
Parasternal long axis (PLAX) M-mode view displaying left atrial depth measurement just inferior to the aorta.

Left atrial depth can be measured vertically at end diastole. To perform this parameter, a PLAX M-mode image should be captured with the yellow line positioned over the LA, which is located inferior to the aorta (Figure 8).

#### 2.3.2 Left ventricle

Although extensively characterized because of the accessibility of imaging the LV, many parameters are inadequately described in literature.

##### 2.3.2.1 Left ventricular outflow length

**FIGURE 9:**
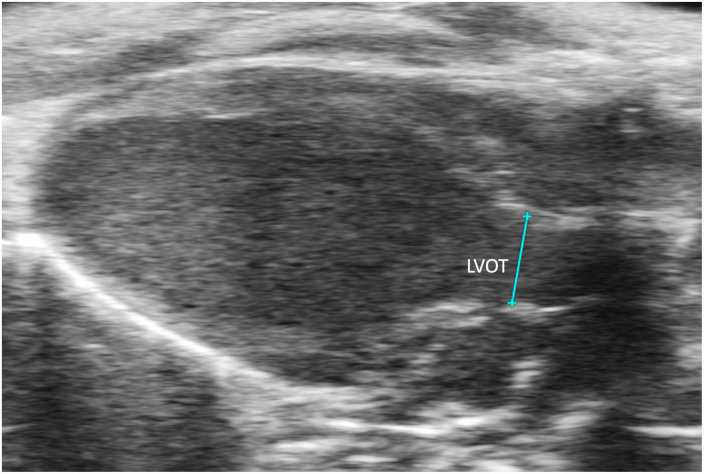
The left ventricular outflow tract (LVOT) length is acquired in the B-mode of the parasternal long axis view (PLAX).

Left ventricular outflow tract length (LVOT) measures, in millimeters, the left ventricular outflow tract, just proximal to the aortic valve, at end diastole. It is measured by selecting “LVOT” under “AoV Flow” in a PLAX B-mode image containing the left ventricular outflow tract (Figure 9). LVOT would be a valuable measure for mouse models of aortic stenosis because it can be used to calculate aortic valve area (AVA) (see below).

##### 2.3.2.2 Left ventricular outflow tract velocity time integral

**FIGURE 10:**
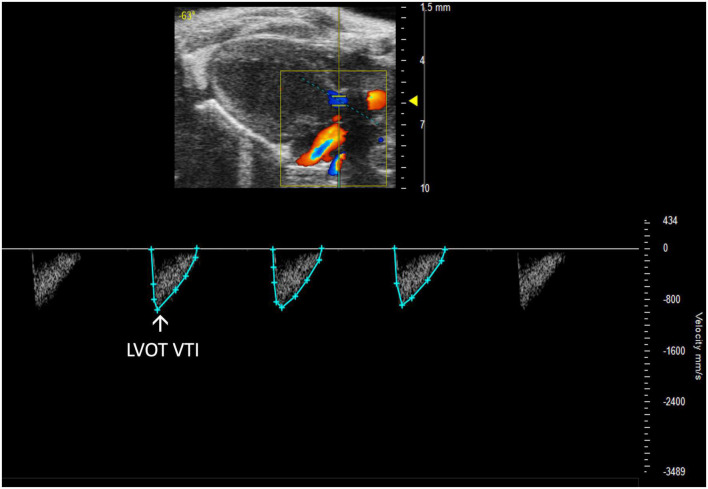
Representative image displaying left ventricular outflow tract velocity time integral (LVOT VTI), a measure of blood flow out of the ascending aorta which is obtained by calculating the area under the curve in the PW Doppler mode of the parasternal long axis view.

Left ventricular outflow tract velocity time integral (LVOT VTI) is defined as a measurement of blood flow out of the ascending aorta. It can be measured as the area under the curve in a PLAX PW Doppler mode image with the cursor over the flow of the ascending aorta (Figure 10). LVOT VTI has been shown to be representative of CO in humans and more predictive than EF or Doppler derived CO in patients with heart failure (10). Thus, we hypothesize that LVOT VTI is an accurate measure of systolic function and CO in mice as well, but this remains an interesting area of study for future directions. LVOT and LVOT VTI can be used to calculate SV, CO, and AVA:


$$L\,V\,O\,T\,S\,V=0.785\times L\,V\,O\,T^{2}\times L\,V\,O\,T\,V\,T\,I$$

$$L\,V\,O\,T\,C\,O=\frac{L\,V\,O\,T\,S\,V\times H\,R\,f\,r\,o\,m\,L\,V\,O\,T}{1000}$$

$$A\,V\,A=\frac{{\left(\frac{L\,V\,O\,T}{2}\right)}^{2}\times \pi \times L\,V\,O\,T\,V\,T\,I\,P\,e\,a\,k\,V\,e\,l}{A\,V\,P\,e\,a\,k\,V\,e\,l}$$

#### 2.3.3 Mitral valve

##### 2.3.3.1 Mitral regurgitation

**FIGURE 11:**
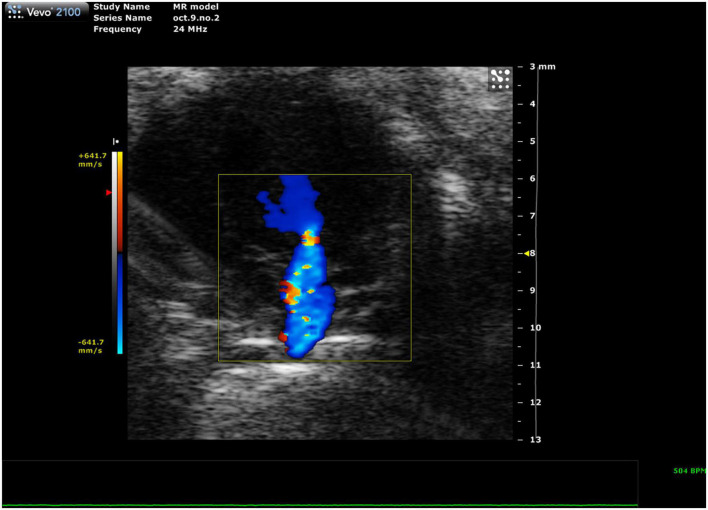
Representative image showing a mixed color pattern of systolic backflow from the left ventricle to the left atrium in severe mitral regurgitation.

**FIGURE 12:**
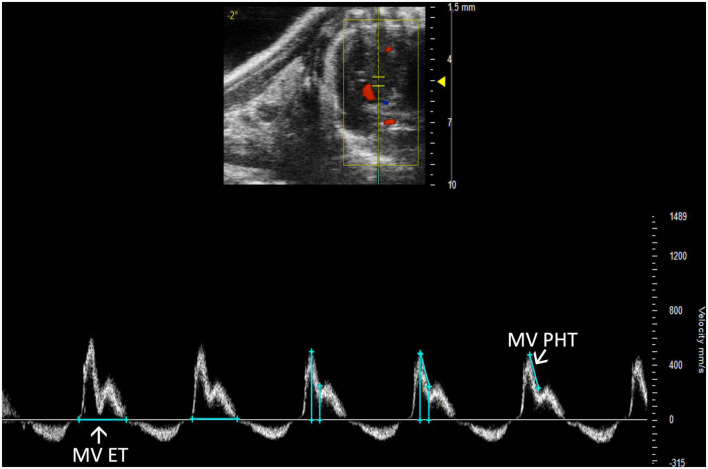
Pulsed wave (PW) Doppler mode image showing mitral valve pressure half time (MV PHT), a measure of mitral stenosis and mitral valve ejection time (MV ET), the duration of diastolic filling in the left ventricle.

Mitral regurgitation (MR) has not been well-described in mice because of their resistance to developing mitral valve prolapse (11). However, Li et al. recently reported the first mouse model of severe MR (Figure 11, 12). To continue development of mouse models of MR, it is important to be able to recognize the presence of mitral regurgitation, which appears as a mixed color pattern of systolic backflow from the LV to the LA on PSAX Color Doppler.

##### 2.3.3.2 Mitral valve pressure half time

Mitral stenosis is another disease that is not well-replicated in mice. However, mitral valve pressure half time (MV PHT) is a potentially useful parameter in the setting of mitral stenosis (10). MV PHT is the time interval, in milliseconds, for the maximum mitral gradient to reduce to half the maximum initial value. It is most accurately measured in a PW Doppler image of the mitral valve by measuring maximum velocity, calculating half of the maximum velocity, and dropping another velocity measurement on the mitral valve curve equal to half of the maximum velocity. Finally, the peaks of the velocity measurements are measured as MV PHT, and the velocity measurements can be deleted (Figure 12).

##### 2.3.3.3 Mitral valve ejection time

Mitral valve ejection time (MV ET) is equal to the duration of diastolic filling of the LV; therefore, can be used to further characterize diastolic dysfunction. MV ET is measured at the end of IVRT to the beginning of IVCT on a PW Doppler of the mitral valve (Figure 12).

#### 2.3.4 Aortic valve

Aortic regurgitation has been seen in existing mouse models; however, methods for analysis beyond visual recognition have not been well-established.

##### 2.3.4.1 Aortic insufficiency deceleration

**FIGURE 13:**
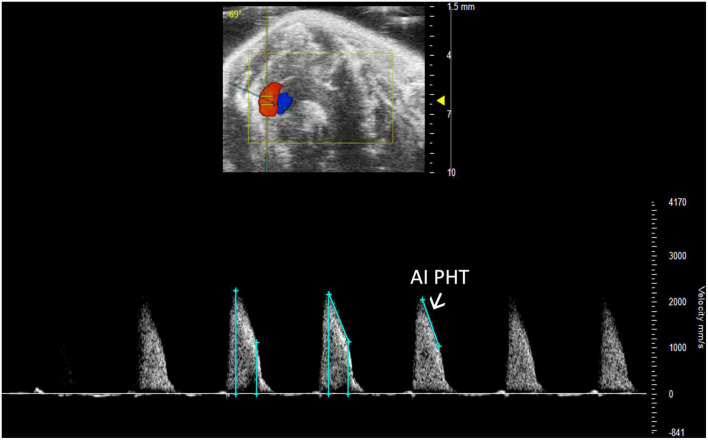
Aortic insufficiency pressure half time (AI PHT) represents the degree of regurgitation and reflects the left ventricular end diastolic pressure and is measured in pulsed wave (PW) Doppler mode of the aortic valve.

Aortic insufficiency pressure half time (AI PHT) is the rate of deceleration of Doppler signal, indicating the degree of regurgitation and LVEDP (13). The same method to measure MV PHT is used to measure AI PHT in PW Doppler mode of the aortic valve (Figure 13). Aortic insufficiency half time is automatically calculated.

##### 2.3.4.2 Aortic acceleration time

**FIGURE 14:**
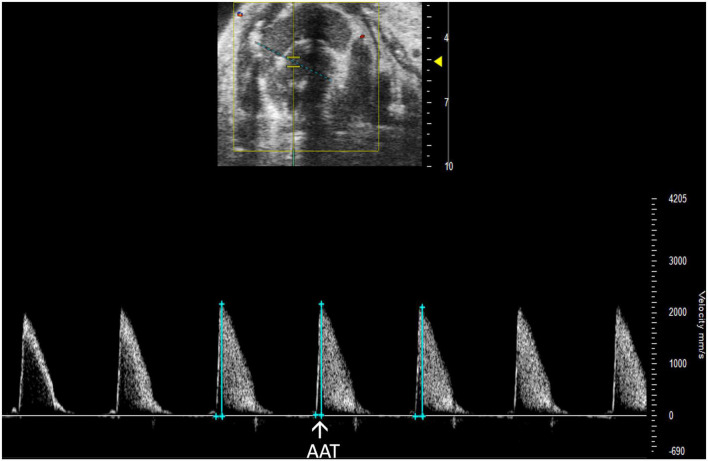
Pulsed wave (PW) Doppler mode image showing analysis of the aortic acceleration time (AAT), a measurement of left ventricular contractility in mice.

Aortic acceleration time (AAT) has been validated by Perez et al. to assess LV contractility in mice. It is measured in PW Doppler mode as the time to peak aortic velocity (Figure 14). Studies in humans suggest that the ratio of AAT to aortic ejection time (AET) predicts aortic stenosis severity (14), although it has not yet been evaluated in mice.

## 3 Anticipated results

Normalizing the acquisition and analysis of infrequently used parameters coupled with commonly used values will provide further insight into both left and right-sided cardiac function. In recent years, the importance of the right heart has become more evident but a complete understanding remains elusive. Implementing the additional parameters described here will generate a more comprehensive picture of cardiovascular disease in mouse models that can be applied to parallel human conditions.

### 3.1 Limitations

All analyses are limited by human error in image acquisition. The RV must be captured in PLAX M-mode in order to measure RV parameters, however, as noted by Brittain et al., sometimes the RV is only visible when dilated. If the transducer is misaligned with blood flow for Doppler modes, blood velocity will be decreased for LVOT VTI and RVOT VTI measurements and SV and CO will be falsely low (3). To obtain the most accurate readings, blood flow direction should be as vertical as possible (up or down) and the dotted line aligned with blood flow in all PW Doppler images. Another limitation is the increased training and effort required to accurately and reproducibly perform a significant number of additional parameters as well as the increased time required to capture the extra views.

## 4 Discussion

In our quest to improve resources, expand and optimize echocardiography parameters in rodent models, we realized a need for protocols and information to define and facilitate infrequently used parameters. Here, we present multiple parameters that are rarely used and provide a thorough description of echocardiography acquisition and analysis of these parameters. Although VisualSonics Vevo imaging systems were used to obtain these images and derive parameters, the same approaches should be applicable to other comparable imaging systems such as S-Sharp (15, 16). In a study that measured the grey scale imaging performance of 17 preclinical transducers over 10 years, all except one S-sharp transducer were Vevo transducers (17), thus, our protocol likely covers the instruments commonly available for use. A summary of these infrequently used parameters combined with common echocardiography parameters as well as data values from 10 to 12-week-old male and female mice from C57BL/6, BalbC, and 129Sv mouse strains can be found in Table 1.

**TABLE 1:** Anatomic structures, modes, definitions, and reference values of both common and infrequently used parameters for the C57BL/6, BalbC, and 129Sv mouse strains.

Anatomic structure	Mode	Parameter	Definition	C57BL/6 (*n* = 11)	BalbC (*n* = 10)	129Sv (*n* = 9)
Mitral valve (MV)	PW Doppler	MV E (mm/s)	Mitral valve early peak	754.47 ± 412.82	939.27 ± 167.53	374.77 ± 126.08
		MV A (mm/s)	Mitral valve atrial peak	556.38 ± 265.92	525.27 ± 244.75	258.80 ± 91.50
		IVRT (ms)	Isovolumetric relaxation time	15.23 ± 4.81	13.31 ± 2.89	20.76 ± 8.83
		IVCT (ms)	Isovolumetric contraction time	10.58 ± 4.37	9.69 ± 2.73	19.24 ± 9.34
		MV VTI (mm)	Velocity time interval of mitral valve inflow	23.10 ± 13.19	32.63 ± 5.69	11.95 ± 4.74
		NFT (ms)	No flow time	71.82 ± 12.79	83.28 ± 9.07	90.61 ± 11.63
		AET (ms)	Aortic ejection time	45.15 ± 7.23	62.64 ± 4.17	49.41 ± 10.39
		MV PHT (ms)	Mitral valve pressure half time	4.31 ± 2.30	7.24 ± 2.32	4.12 ± 0.70
		MV ET (ms)	Mitral valve ejection time	55.74 ± 14.25	72.28 ± 13.02	56.25 ± 9.26
	PW tissue Doppler	E’ (mm/s)	Mitral valve early peak	−18.72 ± 7.47	−22.51 ± 3.95	−18.64 ± 3.47
		A’ (mm/s)	Mitral valve atrial peak	−23.11 ± 12.05	−19.62 ± 5.69	−20.11 ± 3.48
Aortic valve (AoV)	PW Doppler	AV peak (mm/s)	Peak velocity of blood flow through the aortic valve	2537.54 ± 870.16	1024.86 ± 134.84	2265.77 ± 1453.38
		AET (ms)	Aortic ejection time	56.16 ± 13.11	62.64 ± 4.17	49.41 ± 6.46
		AI PHT (ms)	Aortic insufficiency pressure half time	22.65 ± 3.35	30.31 ± 4.84	20.70 ± 4.42
		AAT (ms)	Aortic acceleration time	11.76 ± 2.70	15.24 ± 2.63	10.80 ± 3.17
Pulmonary artery (PA) and valve (PV)	PW Doppler	PV peak (mm/s)	Peak velocity of blood flow through the pulmonic valve	−640.94 ± 164.23	−682.15 ± 141.19	−632.16 ± 240.09
		PAT (ms)	Pulmonic valve acceleration time	18.69 ± 4.25	24.83 ± 4.96	25.46 ± 4.07
		PET (ms)	Pulmonic valve ejection time	58.45 ± 6.95	63.89 ± 4.50	62.64 ± 6.01
		PV VTI (mm)	Velocity time interval of blood through the pulmonic valve	26.20 ± 5.36	32.29 ± 7.04	27.29 ± 12.43
		PR Peak Vel (mm/s)	Maximum velocity of regurgitant stream through the pulmonic valve	149.18 ± 40.46	206.17 ± 135.15	117.32 ± 42.55
		PA VTI (mm)	Velocity time interval of blood flow through the pulmonary artery	29.95 ± 6.65	32.29 ± 7.04	28.24 ± 6.10
	PSLAX B-mode	PV diameter (mm)	Length of the pulmonic valve	1.38 ± 0.18	1.16 ± 0.25	1.42 ± 0.09
Left atrium (LA)	PSLAX M-mode	LA (mm)	Depth of the left atrium	2.04 ± 0.44	1.29 ± 0.34	2.98 ± 0.69
Left ventricle (LV)	PSLAX B-mode	ENDOmajr;s/d (mm)	Length of the LV endocardium during systole and diastole	6.35 ± 0.81/7.28 ± 0.90	6.58 ± 0.68/7.26 ± 0.72	5.88 ± 0.52/6.81 ± 0.53
		EPImajr;s/d (mm)	Length of the LV epicardium during systole and diastole	7.24 ± 0.73/7.94 ± 0.84	7.02 ± 0.71/7.76 ± 0.74	6.68 ± 0.48/7.45 ± 0.58
		LVOT (mm)	Left ventricular outflow tract length	1.58 ± 0.26	1.51 ± 0.10	1.77 ± 0.18
	PSAX B-mode	ENDOarea;s/d (mm)	Area of the LV endocardium during systole and diastole	5.48 ± 3.46/12.40 ± 4.15	7.24 ± 1.72/13.03 ± 2.02	3.94 ± 1.62/9.12 ± 1.56
		EPIarea;s/d (mm)	Area of the LV epicardium during systole and diastole	18.98 ± 5.14/24.76 ± 6.57	17.66 ± 2.64/23.02 ± 3.09	19.06 ± 2.40/22.68 ± 3.31
	PSLAX M-mode	IVS;s/d (mm)	Intraventricular septum during systole and diastole	1.27 ± 0.25/0.81 ± 0.14	0.96 ± 0.16/0.61 ± 0.10	1.19 ± 0.13/0.90 ± 0.16
		LVID;s/d (mm)	Left ventricular internal diameter during systole and diastole	2.35 ± 0.43/3.67 ± 0.31	2.98 ± 0.51/3.91 ± 0.46	1.58 ± 0.22/2.36 ± 0.42
		LVPW;s/d (mm)	Left ventricular posterior wall during systole and diastole	1.21 ± 0.36/0.89 ± 0.32	0.76 ± 0.16/0.65 ± 0.10	1.00 ± 0.14/0.72 ± 0.08
	PSAX M-mode	LVAW;s/d (mm)	Left ventricular anterior wall during systole and diastole	1.52 ± 0.27/0.97 ± 0.18	1.08 ± 0.15/0.69 ± 0.09	1.52 ± 0.40/1.03 ± 0.32
		LVID;s/d (mm)	Left ventricular internal diameter during systole and diastole	2.45 ± 0.70/3.85 ± 0.47	2.99 ± 0.48/3.95 ± 0.39	2.12 ± 0.55/3.37 ± 0.38
		LVPW;s/d (mm)	Left ventricular posterior wall during systole and diastole	1.13 ± 0.22/0.77 ± 0.13	0.75 ± 0.13/0.65 ± 0.11	1.11 ± 0.22/0.80 ± 0.18
	PSLAX PW Doppler	LVOT VTI (mm)	Left ventricular outflow tract velocity time integral	22.27 ± 9.66	16.19 ± 3.08	37.10 ± 18.93
Right ventricle (RV)	PSLAX M-mode	RVID;s/d (mm)	Right ventricular internal diameter during systole and diastole	0.79 ± 0.15 /1.16 ± 0.11	0.99 ± 0.31/1.23 ± 0.31	0.86 ± 0.30 /1.20 ± 0.45
	PSLAX B-mode	RVOT (mm)	Right ventricular outflow tract length	1.46 ± 0.23	1.47 ± 0.20	1.60 ± 0.14
	PSLAX PW Doppler	RVOT VTI (mm)	Right ventricular outflow tract length velocity time integral	18.05 ± 9.83	16.10 ± 7.54	15.38 ± 6.55

Infrequently used parameters are highlighted in gray. Male and female mice at the age of 10–12 weeks of age were used. Data are Mean ± SD.

Previously, the importance of the right heart was underestimated (18). Within the last decade, significant progress has been made in our understanding of the mechanisms involved in acute and chronic right heart failure as well as the role of the pulmonary system (19). However, complete understanding of right heart and pulmonary circuit function remains elusive (18). Exploring right heart and pulmonary function by echocardiography acquisition and analysis of infrequently used parameters such as RVID, RVOT, RVOT VTI, PA VTI, PV Diameter, and PR Peak Velocity in various mouse models may provide more insight into right heart and pulmonary valve dysfunction.

In contrast, left-sided parameters are frequently characterized due to ease of accessibility to the left ventricle during echocardiography acquisition. Despite accessibility, some left-sided parameters including, LA size and depth, LVOT, LVOT VTI, visual assessment of MR and AR, MV PHT, MV ET, AI PHT, and AAT are rarely acquired and analyzed. These values, coupled with frequently used left and right heart parameters, may provide further insight into cardiac function.

This manuscript serves as a guide to understanding the acquisition and analysis of infrequently used echocardiography parameters. With the continued development of mouse models that mimic human cardiac disease it is imperative to define and integrate a complete repertoire of echocardiography parameters to maximize data yields and avoid overlooking elements of unique pathologies.

## Data availability statement

The raw data supporting the conclusions of this article will be made available by the authors, without undue reservation.

## Ethics statement

The animal study was reviewed and approved by IACUC.

## Author contributions

ET, MW, and LS perceived the project. MW and AK acquired the images. ET, MW, AK, and MR analyzed the images. ET and MW drafted the manuscript. All authors revised the manuscript, approved the final version of the manuscript, and agreed to be accountable for all aspects of the work.
